# Partners in crime: two prisoners with foreign body insertion

**DOI:** 10.11604/pamj.2020.36.10.23027

**Published:** 2020-05-08

**Authors:** Androniki Kozana, Gerardina Cavallo

**Affiliations:** 1 Radiology Department, Venizeleion General Hospital of Heraklion, Heraklion, Greece

**Keywords:** Emergency, foreign body, prisoner

## Image in clinical medicine

Two prisoners, a 35-year-old and a 42-year-old man, were admitted to the Emergency Department (ED), following court order. Upon clinical evaluation, their vital signs were normal, while digital rectal examination was suspicious for foreign bodies. Abdominal radiographs were performed and revealed that the first beared rectally two smartphone chargers with their cables (A) and the second a smartphone (B) for communication purposes. Penis pearling is also incidentaly noted (B) not unusual among prisoners to increase intercourse stimulation. Following mild sedation, all rectal foreign bodies were manually extracted and both were discharged in good health after 2-hour monitoring. It is common for prisoners to present with foreign body ingestion or insertion which may be associated with escape or self-destruction attempts, drug-dealing, acts of physical stimulation/assault. Admission may be involuntary and essential history information may be concealed. The emergency physician needs to be vigilant and familiar with the radiographic appearances of commonly encountered foreign bodies.

**Figure 1:**
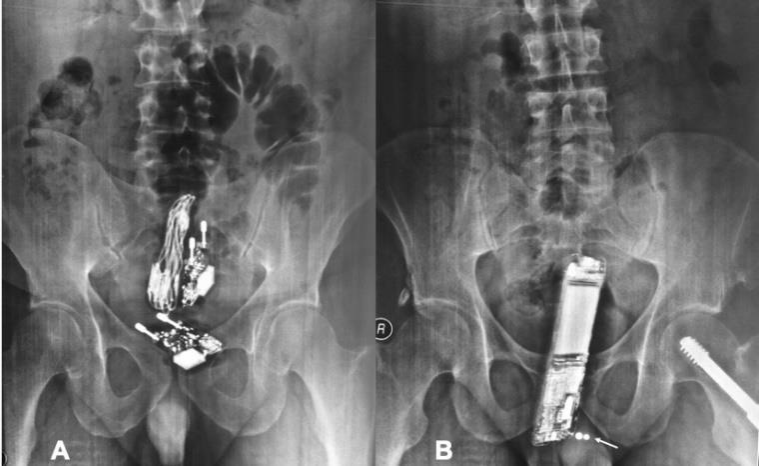
Abdominal x-rays of(A) first prisoner bearing two smartphone chargers rectally; (B) second prisoner bearing a smartphone rectally; incidental penis pearling (arrow)

